# Carbon Nitride Nanosheets as an Adhesive Layer for Stable Growth of Vertically-Ordered Mesoporous Silica Film on a Glassy Carbon Electrode and Their Application for CA15-3 Immunosensor

**DOI:** 10.3390/molecules29184334

**Published:** 2024-09-12

**Authors:** Jun Xing, Hongxin Wang, Fei Yan

**Affiliations:** 1 Shanxi Bethune Hospital, Shanxi Academy of Medical Sciences, Tongji Shanxi Hospital, Third Hospital of Shanxi Medical University, Taiyuan 030032, China; xingjun2022@sxmu.edu.cn; 2 Department of Chemistry, School of Chemistry and Chemical Engineering, Zhejiang Sci-Tech University, Hangzhou 310018, China; 202230107404@mails.zstu.edu.cn

**Keywords:** carbon nitride nanosheets, vertically ordered mesoporous silica film, glassy carbon electrode, electrochemical immunosensor, carbohydrate antigen 15-3

## Abstract

Vertically ordered mesoporous silica films (VMSF) are a class of porous materials composed of ultrasmall pores and ultrathin perpendicular nanochannels, which are attractive in the areas of electroanalytical sensors and molecular separation. However, VMSF easily falls off from the carbonaceous electrodes and thereby impacts their broad applications. Herein, carbon nitride nanosheets (CNNS) were served as an adhesive layer for stable growth of VMSF on the glassy carbon electrode (GCE). CNNS bearing plentiful oxygen-containing groups can covalently bind with silanol groups of VMSF, effectively promoting the stability of VMSF on the GCE surface. Benefiting from numerous open nanopores of VMSF, modification of VMSF’s external surface with carbohydrate antigen 15-3 (CA15-3)-specific antibody allows the target-controlled transport of electrochemical probes through the internal silica nanochannels, yielding sensitive quantitative detection of CA15-3 with a broad detection range of 1 mU/mL to 1000 U/mL and a low limit of detection of 0.47 mU/mL. Furthermore, the proposed VMSF/CNNS/GCE immunosensor is capable of highly selective and accurate determination of CA15-3 in spiked serum samples, which offers a simple and effective electrochemical strategy for detection of various practical biomarkers in complicated biological specimens.

## 1. Introduction

Vertically ordered mesoporous silica films (VMSF) have become increasingly prosperous electrode materials owing to their distinct selectivity at molecular scale and good anti-biofouling properties in complex biological samples [1,2,3,4]. Owing to the isolating nanoporous structure, VMSF-based electrochemical/electrochemiluminescence sensors involve the transport of targets or signal probes along the perpendicular nanochannels and/or biological recognition elements on the outer surface of VMSF [5,6,7,8,9,10,11]. According to the previous reports, indium tin oxide (ITO) coated glasses are very suitable for supporting VMSF and further functionalization of nanomaterials or recognition elements into VMSF’s skeleton [12,13,14,15,16]. However, in comparison with commonly used carbonaceous electrodes (such as glassy carbon electrode (GCE) and three-dimensional graphene), ITO electrodes as the supporting substrate have shortcomings, including slow electron transport properties for small organic molecules and a narrow electrochemical window, which limit the analytical performances of VMSF-based sensors in practical analysis [17]. On account of the instability of VMSF on the carbonaceous electrodes, a group of nanomaterials have been employed to confer carbonaceous electrodes with adhesive properties for stable growth of VMSF, such as silane molecules [18], two-dimensional graphene nanosheets, and their hybrid materials [19,20,21]. In addition, pre-activation of carbonaceous electrodes by simple electrochemical procedure generates the oxygen-containing groups on the electrode [22,23], which is capable of stable fabrication of VMSF [24,25,26,27]. Therefore, developing diverse adhesive layers for stable fabrication of VMSF on the carbonaceous electrode surface is of great importance for extending the practical analytical applications of VMSF.

Ultrathin carbon nitride nanosheets (CNNS) possessing a two-dimensional (2D) graphene-like structure display unique characteristics, including large surface areas, excellent electron transport ability, and good catalysis properties, compared with their bulk counterparts [28]. Nowadays, CNNS has received tremendous research interest and shown significant promise in different applications, such as imaging, sensing, and biotherapy [29]. Moreover, CNNS, usually prepared by exfoliating bulk graphitic-phase carbon nitrides, can provide hydroxy groups for covalently binding with silanol groups of VMSF.

In this paper, CNNS is introduced on the GCE surface via π-π interaction using a simple drip-coating method, and its hydroxyl groups offer a suitable microenvironment for further stable fabrication of VMSF through covalent binding between silanol groups of VMSF and hydroxyl groups of CNNS. Such obtained VMSF/CNNS composite on the GCE has good stability and solves the problem of falling off of VMSF from GCE. Such VMSF/CNNS/GCE is fit for the construction of electrochemical biosensors. As a proof of concept, carbohydrate antigen 15-3 (CA15-3) is usually used as the indicator for lung cancer and breast cancer, which is selected as the analyte to examine the potential application capacity of VMSF/CNNS/GCE. In general, the normal CA15-3 amount in human serum is less than 30 U/mL, and an increased amount indicates an increased risk of developing cancer [30]. Quantitative detection of CA15-3 is realized by modification of CA15-3-specific antibody on the external surface of VMSF. The current signal variation of electrochemical probes (potassium ferricyanide/potassium ferrocyanide, [Fe(CN)_6_]^3−/4−^) after incubation of different concentrations of CA15-3 at the immunosensing interface is related to the CA15-3 concentration. Compared with traditional methods, our proposed electrochemical immunosensor has some advantages of low cost, rapid detection time, and convenient operation. Moreover, the VMSF/CNNS/GCE-based immunosensor has the same anti-biofouling characteristic as the VMSF-modified electrode, which offers a convenient sensing approach for monitoring the amount of CA15-3 and early screening of malignant tumors.

## 2. Results and Discussion

### 2.1. Fabrication of VMSF/CNNS/GCE-Based Immunosensor for Electrochemical Detection of CA15-3

**Scheme 1:**
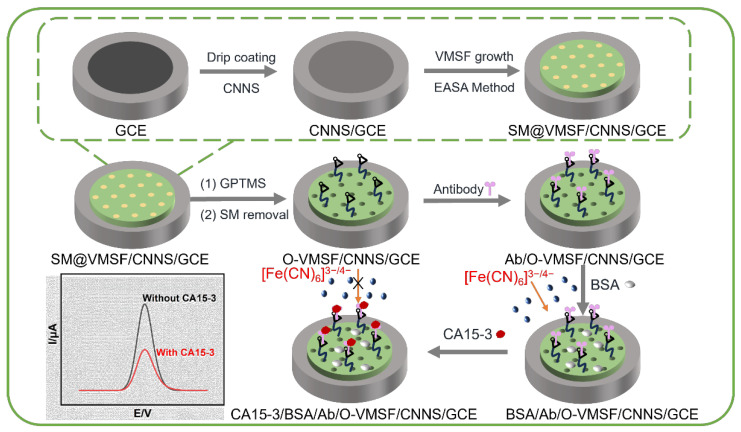
Schematic illustration for the preparation of the VMSF/CNNS/GCE-based immunosensor and the electrochemical determination of CA15-3 with the help of electrochemical probes ([Fe(CN)_6_]^3−/4−^) in solution.

VMSF consists of two regions, namely external surface and inner nanochannels, which can be used for functionalization of active groups and for mass transport of signal probes, respectively. Scheme 1 illustrates the specific construction procedures of CA15-3 immunosensors based on the VMSF/CNNS/GCE. As seen, CNNS is first dripped-coated on the commercial GCE surface through π-π interaction, and the EASA method is then conducted for the growth of VMSF using CNNS/GCE as the working electrode. In the growth process of VMSF, CNNS bearing negative charges and oxygen-containing groups is suitable for electrostatic adsorption of cationic surfactant micelles (SM) and formation of O-Si-O chemical bonds between CNNS and VMSF, exhibiting a good potential adhesive layer for stable growth of VMSF on GCE. When reaction ceases, templated SM are retained inside the nanospaces formed by VMSF’s nanochannels, termed as SM@VMSF/CNNS/GCE. GPTMS carrying both epoxy groups and silane groups is introduced onto the external surface of SM@VMSF/CNNS/GCE, and SM is then extracted from the nanochannels, effectively guaranteeing the epoxy groups distributed on the external surface of VMSF/CNNS/GCE (O-VMSF/CNNS/GCE). Anti-CA15-3 antibody (Ab) enabling specifically recognition of CA15-3 is anchored on the surface of O-VMSF/CNNS/GCE through the chemical reaction between epoxy groups and amino groups. Electrochemical immunosensing interface, namely BSA/Ab/O-VMSF/CNNS/GCE, is obtained after the blocking of non-specific adsorption by BSA. The quantitative mechanism for CA15-3 relies on the current variation of electrochemical probes ([Fe(CN)_6_]^3−/4−^) produced by target CA15-3 binding on the BSA/Ab/O-VMSF/CNNS/GCE. Target CA15-3 and its corresponding antibody can form immunocomposite on the VMSF surface, influencing the access of [Fe(CN)_6_]^3−/4−^ through the silica nanochannels to reach the underlying CNNS/GCE and finally resulting in the declined electrochemical current signals.

### 2.2. Characterization of CNNS

**Figure 1:**
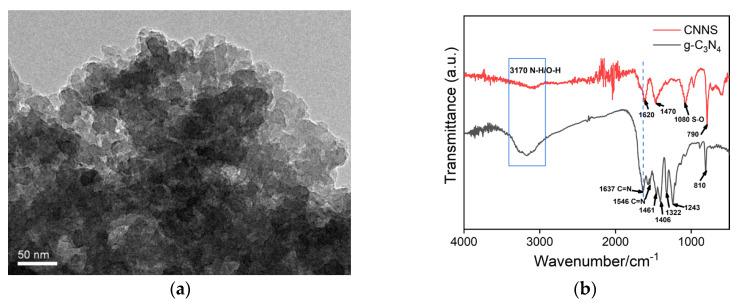
(**a**) TEM images of CNNS. (**b**) FT−IR spectra of g−C_3_N_4_ and CNNS.

TEM were first used to show the morphology of the prepared CNNS. As shown in Figure 1a, CNNS prepared by H_2_SO_4_ exfoliation of bulk g-C_3_N_4_ has a 2D lamellar structure. Figure 1b compares the FT-IR spectra of g-C_3_N_4_ and CNNS. g-C_3_N_4_ displays several characteristic peaks of heterocycle skeleton. The peaks at around 1637 cm^−1^ and 1546 cm^−1^ in correspond to the stretching vibrations of C=N. The characteristic peaks at 1461 cm^−1^, 1406 cm^−1^, 1322 cm^−1^, 1243 cm^−1^, and 1205 cm^−1^ are assigned to the stretching vibrations of C−N. The peak at 810 cm^−1^ is attributed to the typical characteristic peak of the triazine ring. After H_2_SO_4_ treatment, all above characteristic peaks of CNNS have no obvious change, suggesting the remained skeleton of g-C_3_N_4_ heterocycle. The new peak at 1080 cm^−1^ resulted from the S-O stretching in SO_4_^2−^, showing the acidified effect of H_2_SO_4_ for g-C_3_N_4_. Moreover, compared to g-C_3_N_4_, the adsorption peak of CNNS at ~1600 cm^−1^ shifts slightly to the low wavenumber, suggesting the existence of carboxylate groups. The broad band at 3170 cm^−1^ belongs to the stretching vibrations of N-H and O-H. The above data indicate that CNNS has a 2D planar structure with oxygen-containing groups, which is suitable for improved stability of VMSF on the GCE surface.

### 2.3. Characterization of VMSF

**Figure 2:**
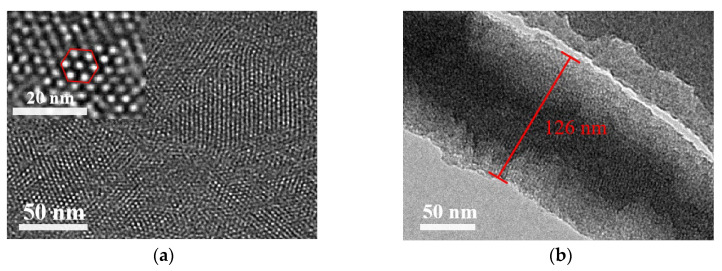

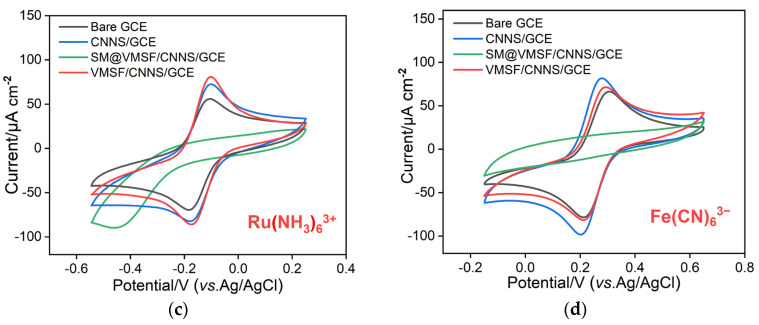
TEM images of VMSF: (**a**) top view and (**b**) cross−sectional view. CV curves of bare GCE, CNNS/GCE, SM@VMSF/CNNS/GCE and VMSF/CNNS/GCE in 0.05 M KHP containing 0.5 mM Ru(NH_3_)_6_^3+^ (**c**) and 0.5 mM Fe(CN)_6_^3−^ (**d**).

TEM images show that the fabricated VMSF on CNNS/GCE surface is characteristic of numerous regularly aligned nanopores (Figure 2a) and nanochannels parallel to each other (Figure 2b). By measurement, the diameter and thickness of VMSF are 2~3 nm and 110 nm, respectively, which is similar to those of VMSF prepared on the other substrates (e.g., ITO and gold electrodes) [31,32,33]. Moreover, electrochemical technique (cyclic voltammetry (CV)) was used to provide information about intactness and mass transport ability for electrochemical probes of the fabricated VMSF/CNNS/GCE. As presented in Figure 2c,d, enhanced redox peak currents for both Ru(NH_3_)_6_^3+^ and Fe(CN)_6_^3−^ are found at the CNNS/GCE, compared with bare GCE, indicating the good conductivity of synthesized CNNS. No Faradic currents of these two electrochemical probes can be seen at the SM@VMSF/CNNS/GCE, which is due to the impeded effect of SM confined into the silica nanochannels and further suggests the integrity of as-synthesized VMSF on the CNNS/GCE surface. By comparing the electrochemical current variation of two charged electrochemical probes before and after exclusion of SM from silica nanochannels, VMSF on the CNNS/GCE surface also exhibits its inherent charge permselectivity, namely amplifying the signal of positively charged Ru(NH_3_)_6_^3+^ and suppressing the signal of negatively charged Fe(CN)_6_^3−^.

### 2.4. Feasibility of BSA/Ab/O-VMSF/CNNS/GCE Immunosensor for Detection of CA15-3

**Figure 3:**
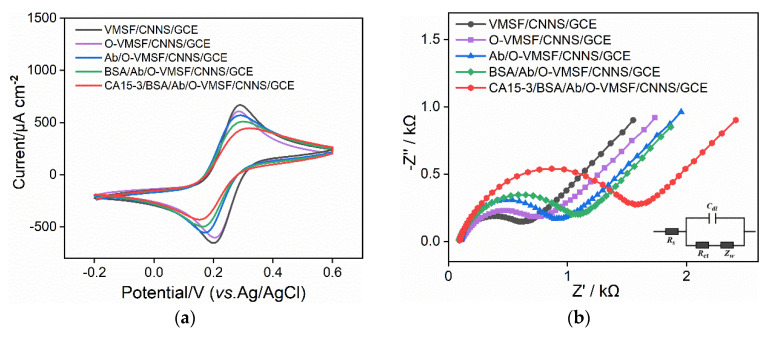
CV (**a**) and EIS curves (**b**) obtained during the fabrication procedure of the BSA/Ab/O−VMSF/CNNS/GCE immunosensor in 0.1 M KCl containing 2.5 mM [Fe(CN)_6_]^3−/4−^. The concentration of CA15−3 is 10 U/mL. Inset in (**b**) is the equivalent circuit diagram.

CV and EIS are two kinds of commonly used techniques for characterization of electrode construction. As shown in Figure 3a, dropwise modification of GPTMS, Ab and BSA on the VMSF/CNNS/GCE can lead to the sequentially diminished electrochemical current signals of [Fe(CN)_6_]^3−/4−^, which arise from the hindered transport of [Fe(CN)_6_]^3−/4−^ through the silica nanochannels to the underlying GCE. Thanks to the immunocomposite consisting of CA15-3 and Ab formed at the sensing interface, the fabricated BSA/Ab/O-VMSF/CNNS/GCE immunosensor is used to detect 10 U/mL CA15-3, giving rise to the decreased electrochemical current signals of [Fe(CN)_6_]^3−/4−^ and indicating the successful fabrication of electrochemical immunosensor. Figure 3b displays the corresponding EIS plots, revealing the interfacial properties during the fabrication procedure of immunosensor. With the dropwise incubation of GPTMS, Ab, BSA, and CA15-3 on VMSF/CNNS/GCE, the electrode surface is covered with non-conductive substances, leading to a gradual increase in electron transfer resistance (R_ct_) at the electrode/electrolyte interface, as evidenced by the increasing diameter of semicircle diameter in the high-frequency region (Figure 3b). EIS curves are fitted by equivalent circuit model shown in the inset of Figure 3b and the obtained R_ct_ values at the VMSF/CNNS/GCE, Ab/O-VMSF/CNNS/GCE, BSA/Ab/O-VMSF/CNNS/GCE and CA15-3/BSA/Ab/O-VMSF/CNNS/GCE are 614 Ω, 747 Ω, 843 Ω, 1055 Ω and 1543 Ω, respectively. The change trend in electron transfer resistance presented in Figure 3b is in accordance with the current responses shown in Figure 3a, further proving the feasibility of the designed BSA/Ab/O-VMSF/CNNS/GCE immunosensor for CA15-3 determination.

### 2.5. Optimization of Experimental Conditions for CA15-3 Detection Using BSA/Ab/O-VMSF/CNNS/GCE Immunosensor

**Figure 4:**
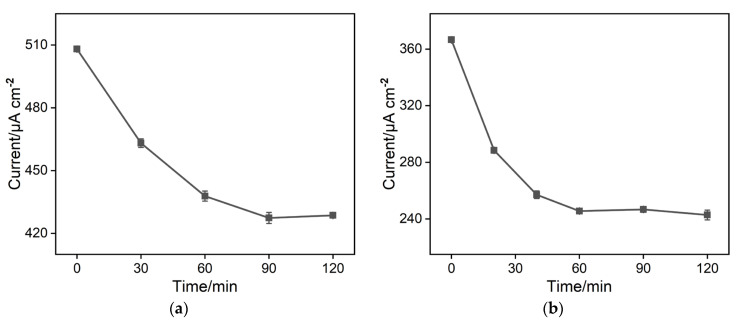
(**a**) Electrochemical current signals of 2.5 mM [Fe(CN)_6_]^3−/4−^ at the O−VMSF/CNNS/GCE after incubation with CA15−3 antibody at various incubation times. (**b**) Electrochemical current signals of 2.5 mM [Fe(CN)_6_]^3−/4−^ at the BSA/Ab/O−VMSF/CNNS/GCE immunosensor after incubation with 1 U/mL CA15−3 at various incubation times. The supporting electrolyte is 0.1 M KCl solution, and the error bars represent the relative standard deviation (RSD) of three measurements.

The amount of CA15-3 antibody immobilized at the electrode surface can affect the analytical performance of CA15-3 determination. Therefore, incubation times for CA15-3 antibody and CA15-3 at the O-VMSF/CNNS/GCE and BSA/Ab/O-VMSF/CNNS/GCE, respectively, were optimized, and the results are shown in Figure 4. As seen, electrochemical current signals of [Fe(CN)_6_]^3−/4−^ obviously decrease and reach the plateau after a period of time. Therefore, the optimal incubation times for CA15-3 antibody and CA15-3 are 90 min and 60 min, respectively.

### 2.6. Electrochemical Detection of CA15-3 Using the Immunosensor

**Figure 5:**
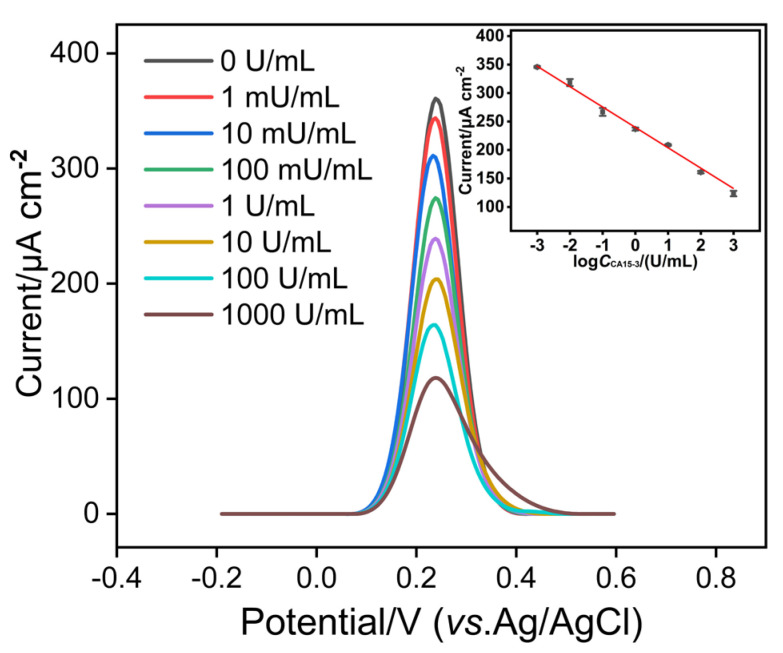
DPV responses of the prepared BSA/Ab/O−VMSF/CNNS/GCE to various concentrations of CA15−3 in 0.1 M KCl solution containing 2.5 mM [Fe(CN)_6_]^3−/4−^. Inset is the corresponding calibration curve for the detection of CA15−3. The error bars represent the RSD of three measurements.

BSA/Ab/O-VMSF/CNNS/GCE was used to incubate with various concentrations of CA15-3 and the obtained CA15-3/BSA/Ab/O-VMSF/CNNS/GCE were tested in 0.1 M KCl containing 2.5 mM [Fe(CN)_6_]^3−/4−^. As depicted in Figure 5, the tested anodic peak current is proportional to the CA15-3 concentration in the range from 1 mU/mL to 1000 U/mL, giving rise to a good linear fitting equation of *I* (μA cm^−2^) = –35.8 log*C*_CA15-3_ (U/mL) + 240 (*R*^2^ = 0.995). The same trend is shown in CV and EIS data (Figure S1), but lower variation is obtained, suggesting the sensitive DPV technique. The limit of detection (LOD) for CA15-3 was calculated to be 0.47 mU/mL, which was lower than most of the electrochemical immunosensors reported previously (Table 1). Moreover, the fabrication of CA15-3/BSA/Ab/O-VMSF/CNNS/GCE has advantages of convenient operation and economic cost.

**Figure 6:**
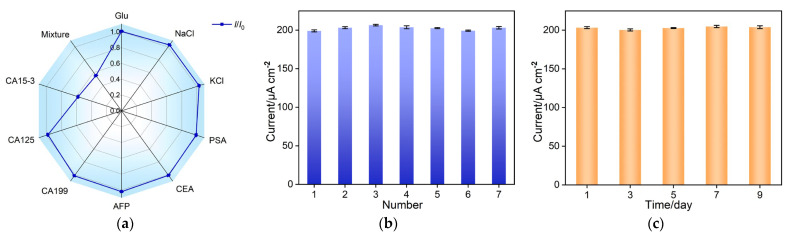
(**a**) Selectivity of the fabricated BSA/Ab/O−VMSF/CNNS/GCE immunosensor: electrochemical responses of BSA/Ab/O−VMSF/CNNS/GCE to interference substances (100 ng/mL PSA, 10 U/mL CA19−9, 10 mU/mL CA125, 100 ng/mL AFP, 0.1 ng/mL CEA, 100 μM NaCl, 100 μM KCl, and 100 μM Glu), CA15−3 (10 U/mL) and their mixture. *I*_0_ and *I* refer to the electrochemical signals obtained at the BSA/Ab/O−VMSF/CNNS/GCE immunosensor before and after incubation with interfering substances, target CA15−3 or their mixture, respectively. (**b**) Reproducibility of the seven BSA/Ab/O−VMSF/CNNS/GCE immunosensors prepared in different batches after incubation with 10 U/mL of CA15−3. (**c**) Stability of the developed BSA/Ab/O−VMSF/CNNS/GCE immunosensor to 10 U/mL CA15−3 after storage for different days. The detection solution in (**a**–**c**) is 0.1 M KCl solution containing 2.5 mM [Fe(CN)_6_]^3−/4−^ and error bars represent the standard deviations of three measurements.

To evaluate the analytical performance of the fabricated BSA/Ab/O-VMSF/CNNS/GCE immunosensor for CA15-3 detection, several important indicators, including selectivity, reproducibility, and stability, were studied. As shown in Figure 6a, various biomarkers (PSA, CA19-9, CA125, AFP, and CEA) and inorganic cations (Na^+^ and K^+^) were determined by BSA/Ab/O-VMSF/CNNS/GCE, and they could not produce obvious electrochemical responses. Only when incubated with target CA15-3 or a mixture consisting of CA15-3 and above interfering species are remarkably decreased electrochemical responses found at the BSA/Ab/O-VMSF/CNNS/GCE, indicating the good anti-interference and selectivity for CA15-3 detection. To verify the reproducibility of the designed BSA/Ab/O-VMSF/CNNS/GCE, seven BSA/Ab/O-VMSF/CNNS/GCE were prepared in different batches under the same procedures and incubated with 10 U/mL CA15-3 (Figure 6b), giving rise to comparable electrochemical responses with an RSD value of 1.3%. Figure 6c shows the storage stability of BSA/Ab/O-VMSF/CNNS/GCE for CA15-3 detection within a week, yielding ignorable variation on the electrochemical responses with an RSD of 0.8%. Excellent reproducibility and stability of BSA/Ab/O-VMSF/CNNS/GCE are revealed in Figure 6b,c, implying the great potential of the proposed BSA/Ab/O-VMSF/CNNS/GCE for real sample analysis.

### 2.7. Real Sample Analysis

The standard addition method was employed to assess the practical potential of the BSA/Ab/O-VMSF/CNNS/GCE immunosensor in fetal bovine serum. Fetal bovine serum is used as a model for “real sample” and first subjected to simple dilution using 0.01 M PBS (pH 7.4) and then spiked with several known concentrations of CA15-3, followed by quantitative determination by our fabricated BSA/Ab/O-VMSF/CNNS/GCE. Recovery and RSD are two important indexes to examine the potential analytical performance of the proposed electrochemical immunosensor in real samples. Recovery is defined as the ratio of the detected concentration by our immunosensor to the spiked known concentration. RSD indicates the degree of dispersion or consistency of three tested results under the same experimental conditions. Low RSD confirms the high precision of the sensors. The results in Table 2 suggest that RSD values obtained from these samples are below 1.9% and recoveries are in the range of 100~106%. Therefore, our proposed BSA/Ab/O-VMSF/CNNS/GCE shows good reliability, which is suitable for analysis of CA15-3 in real samples.

## 3. Materials and Methods

### 3.1. Chemicals and Materials

Carbohydrate antigen 15-3 (CA15-3), anti-CA15-3 antibody, carcinoembryonic antigen (CEA), alpha-fetoprotein (AFP), carbohydrate antigen 125 (CA125), carbohydrate antigen 19-9 (CA19-9), and fetal bovine serum were purchased from Beijing KeyGen Biotech Co., Ltd. (Beijing, China). Prostate-specific antigen (PSA) was procured from Beijing Biodragon Immunotechnologies Co., Ltd. (Beijing, China). Tetraethyl orthosilicate (TEOS, 98%), 3-glycidoxypropyltrimethoxysilane (GPTMS, 97%), cetyltrimethylammonium bromide (CTAB, 99%), bovine serum albumin (BSA), sodium dihydrogen phosphate dih7ydrate (NaH_2_PO_4_·2H_2_O, 99%), disodium hydrogen phosphate dodecahydrate (Na_2_HPO_4_·12H_2_O, 99%), glucose (Glu, 100%), sodium hydroxide (NaOH, 97%), sodium chloride (NaCl, 99.5%), potassium chloride (KCl, 99.5%), potassium ferricyanide (K_3_Fe(CN)_6_, 99.5%), and potassium ferrocyanide (K_4_Fe(CN)_6_, 99.5%) were purchased from Shanghai Aladdin Bio-Chem Technology Co., Ltd. (Shanghai, China). Sulfuric acid (H_2_SO_4_, 98%), acetone (99.5%), anhydrous ethanol (99.8%), and concentrated hydrochloric acid (HCl, 36–38%) were obtained from Hangzhou Shuanglin Reagent Co., Ltd. (Hangzhou, China). Melamine (99%) was purchased from Jiangsu Yonghua Fine Chemicals Co., Ltd. (Suzhou, China) Phosphate-buffered saline (PBS, 0.01 M, pH = 7.4) was prepared using NaH_2_PO_4_·2H_2_O and Na_2_HPO_4_·12H_2_O. All the aqueous solutions used here were prepared using ultrapure water (18.2 MΩ cm) from Milli-Q Systems (Millipore Inc., Burlington, MA, USA). All chemical reagents were of analytical grade.

### 3.2. Characterizations and Instrumentations

The morphological structures of g-C_3_N_4_, CNNS, and VMSF were characterized using transmission electron microscopy (TEM, model HT7700, Hitachi, Tokyo, Japan). To prepare TEM samples, the VMSF layer was carefully scraped off from the electrode using a scalpel and dispersed in anhydrous ethanol with subsequent ultrasonic dispersion. Subsequently, the resulting dispersion was dripped onto a copper grid. Before morphology characterization under 200 kV, the sample was allowed to air dry naturally. All electrochemical experiments, including cyclic voltammetry (CV), electrochemical impedance spectroscopy (EIS), and differential pulse voltammetry (DPV), were conducted on an Autolab electrochemical workstation (model PGSTAT302N, Metrohm Autolab, Herisau, Switzerland). A conventional three-electrode system was employed, with an Ag/AgCl as the reference electrode, a platinum wire as the counter electrode, and the modified electrode as the working electrode. The frequency range for EIS measurements was from 0.1 Hz to 100 kHz, with a perturbation amplitude of 5 mV. Fourier transform infrared spectroscopy (FT-IR) was measured using a Vertex 70 spectrometer (Bruker, Billerica, MA, USA) through the KBr tablet method.

### 3.3. Preparation of VMSF/CNNS/GCE

The g-C_3_N_4_ was prepared by thermal polycondensation of melamine [39]: 5 g of melamine was weighed and placed in a crucible, heated to 520 °C at the rate of 6 °C per minute in an air atmosphere, calcined at this temperature for 4 h, and ground into a yellow solid powder to obtain g-C_3_N_4_. CNNS was prepared by stripping g-C_3_N_4_ with H_2_SO_4_. Generally, 2 g of g-C_3_N_4_ powder is added to 40 mL of H_2_SO_4_ (98 wt%) and stirred at room temperature for 10 h. Then, the mixture was slowly poured into 100 mL of deionized water and put under ultrasound for 8 h. After pouring out the clear supernatant, the obtained precipitate was repeatedly washed with deionized water by centrifugation at 12,000 rpm, and a stable colloidal suspension of CNNS was obtained. Finally, CNNS powder was obtained by freeze-drying.

GCE was polished by alumina powder with specifications of 0.5 μm, 0.3 μm, and 0.05 μm, successively. Then, ultrasonic cleaning GCE with anhydrous ethanol (99.7%) and ultrapure water in turn. 10 μL of CNNS colloid was dropped on the polished GCE and dried at 60 °C to obtain CNNS/GCE, and then VMSF was grown by the electrochemically assisted self-assembly (EASA) method [40,41,42]. Preparation of precursor solution containing silica: addition of 3050 μL TEOS into the mixed solution of 20 mL ethanol, 20 mL NaNO_3_ (0.1 M, pH = 2.6) and 1.585 g CTAB. Then, the mixed solution was vigorously stirred for 2.5 h. After stirring, a three-electrode system was used, with CNNS/GCE as the working electrode, and a constant voltage of −2.2 V was applied for electrodeposition for 5 s. After that, the electrode was quickly taken out and washed with a large amount of ultrapure water, dried by nitrogen gas, and aged at 80 °C overnight. At this time, the silica nanopores contained surfactant micelles (SM), named SM@VMSF/CNNS/GCE. Due to the hydrophobicity of SM, SM@VMSF/CNNS/GCE was immersed in an HCl-ethanol solution (0.1 M) and stirred for 5 min to remove micelles, yielding VMSF/CNNS/GCE with open channels.

### 3.4. Fabrication of the VMSF/CNNS/GCE-Based Immunosensor

To immobilize the anti-CA15-3 antibody on the outer surface of the VMSF/CNNS/GCE, a bifunctional reagent GPTMS containing both epoxy groups and silane groups was selected as the crosslinking agent. SM@VMSF/CNNS/GCE was immersed in GPTMS (2.26 mM in ethanol) for 30 min, and epoxy groups were introduced on the outer surface of VMSF instead of internal nanochannels. After washing with ultrapure water, immersing in a solution containing 0.1 M HCl/ethanol, and stirring for 5 min to remove SM, the O-VMSF/CNNS/GCE with open inner nanochannels and epoxy groups on the external surface was obtained.

Then, 10 μL anti-CA15-3 antibody solution (1 μg/mL in 0.01 M PBS, pH = 7.4) was dripped on the surface of the O-VMSF/CNNS/GCE and incubated at 4 °C for 90 min. Afterwards, the electrode was washed with PBS (0.01 M, pH = 7.4) to remove unbound anti-CA15-3 antibodies on the electrode surface, obtaining Ab/O-VMSF/CNNS/GCE. BSA (1 wt%) solution was utilized to incubate with Ab/O-VMSF/CNNS/GCE at 4 °C for 30 min to block nonspecific sites. After being washed with ultrapure water, the obtained immunosensing electrode was denoted as BSA/Ab/O-/VMSF/CNNS/GCE.

### 3.5. Electrochemical Detection of CA15-3

The BSA/Ab/O-VMSF/CNNS/GCE immunosensor was incubated with different concentrations of CA15-3 at 4 °C for 60 min, respectively. The detection solution was 2.5 mM [Fe(CN)_6_]^3−/4−^ in 0.1 M KCl solution. DPV was used to measure the electrochemical signal of the BSA/Ab/O-VMSF/CNNS/GCE immunosensor before and after CA15-3 binding. Without complicated pretreatment, the fetal bovine serum as an actual sample was used to simulate the medium of actual human serum and diluted 50 times with PBS (0.01 M, pH = 7.4) buffer solution and directly used for the recovery experiment using the standard addition method.

## 4. Conclusions

In summary, through the introduction of the adhesive GNNS, stability of VMSF on GCE surface was significantly promoted, and application of such VMSF/GNNS nanocomposite on GCE for construction of immunosensors had been studied. Due to the oxygen-containing groups of CNNS, Si-O-Si chemical bonds between VMSF and CNNS can be formed, which ensures the stable growth of VMSF on the GCE surface and greatly improves the accuracy and reproductivity of the electroanalytical sensor. Owing to the plentiful open nanopores of VMSF, VMSF possesses good permeability for transport of electrochemical probes ([Fe(CN)_6_]^3−/4−^). When the CA15-3 antibody is anchored on the external surface of VMSF and incubated with target CA15-3, access of [Fe(CN)_6_]^3−/4−^ to the underlying GCE through silica nanochannels is blocked, generating the declined electrochemical current and finally enabling the quantitative detection of CA15-3. A broad detection range of 1 mU/mL to 1000 U/mL and a low LOD of 0.47 mU/mL are achieved by our fabricated VMSF/CNNS/GCE-based immunosensor. Furthermore, determination of CA15-3 in spiked fetal bovine serum samples was used to evaluate the potential practical application of the fabricated immunosensor, displaying good selectivity and accuracy. This immunosensor extends the adhesive layer for growth of VMSF on GCE surface and also offers a simple and effective electrochemical strategy for detection of various practical biomarkers in complicated biological specimens. To better meet the demand of rapid determination in clinical applications, miniaturization and intellectualization of our fabricated electrochemical immunosensor require improvement in future research by combination with microelectronics technology and smart phones.

## Figures and Tables

**Table 1:** Analytical performances of various electrochemical sensors for detection of CA15-3.

Materials	Method	Liner Range (U/mL)	LOD (mU/mL)	Refs.
BSA/Ab/Ag/TiO_2_/rGO/GCE	CA	0.1–300	70	[34]
BSA/Ab/DAP-AuNPs/P3ABA/2D-MoSe_2_/GO/SPCE	DPV	0.14–500	140	[35]
EA/Ab/CuS-RGO/SPE	DPV	1–150	300	[36]
MCH/Ab/CoS_2_-GR-AuNPs/SPE	DPV	0.1–150	30	[37]
BSA/Ab/Au@Pt NCs/Fc-g-CS/GCE	DPV	0.5–200	170	[38]
BSA/Ab/O-VMSF/CNNS/GCE	DPV	0.001–1000	0.47	this work

rGO: reduced graphene oxide; CA: chronoamperometry; DAP: deposited redox dye; P3ABA: poly(3-aminobenzylamine); GO: graphene oxide; SPCE: screen-printed carbon electrode; DPV, differential pulse voltammetry; EA: ethanolamine; SPE: screen printed electrode; MCH: 6-Mercapto-1-hexanol; GR: graphene; Au@Pt NCs: dendritic Au@Pt core–shell nanocrystals; Fc-g-CS: ferrocene grafted with chitosan.

**Table 2:** Determination of CA15-3 in fetal bovine serum.

Sample	Added ^b^ (ng/mL)	Found (ng/mL)	Recovery (%)	RSD (%, *n* = 3)
serum ^a^	0.0100	0.0100	100	1.9
	1.00	1.06	106	1.1
	100	103	103	0.66

^a^ Fetal bovine serum sample detected in this study is diluted by a factor of 50 using PBS (0.01 M, pH 7.4). ^b^ The concentration of CA15-3 indicated in this Table is obtained after the dilution.

## Data Availability

The data presented in this study are available on request from the corresponding author.

## Supplementary Materials

The following supporting information can be downloaded at: https://www.mdpi.com/article/10.3390/molecules29184334/s1. Figure S1: CV (a) and EIS responses (b) of the prepared BSA/Ab/O-VMSF/CNNS/GCE to various concentrations of CA15-3 in 0.1 M KCl solution containing 2.5 mM [Fe(CN)_6_]^3−/4−^. Inset in (b) is the equivalent circuit diagram.
