# Supplementary material for: Carbon Nitride Nanosheets as an Adhesive Layer for Stable Growth of Vertically-Ordered Mesoporous Silica Film on a Glassy Carbon Electrode and Their Application for CA15-3 Immunosensor
Source: Molecules. 2024 Sep 12;29(18):4334. doi: 10.3390/molecules29184334 (PMC11434449; doi:10.3390/molecules29184334)
Supplement: Supplementary file 1 [file molecules-29-04334-s001.zip › molecules-3080575-supplementary.pdf]

Supporting Information to

Carbon Nitride Nanosheets as an Adhesive Layer for Stable Growth of Vertically-Ordered Mesoporous Silica Film on a Glassy Carbon Electrode and Their Application for CA15-3 Immunosensor

Jun Xing [1], Hongxin Wang [2] and Fei Yan [2,*]

[1] Shanxi Bethune Hospital, Shanxi Academy of Medical Sciences, Tongji Shanxi Hospital, Third Hospital of Shanxi Medical University, Taiyuan 030032, China; xingjun2022@sxmu.edu.cn

[2] Department of Chemistry, School of Chemistry and Chemical Engineering, Zhejiang Sci-Tech University, Hangzhou 310018, China; 202230107404@mails.zstu.edu.cn

* Correspondence: yanfei@zstu.edu.cn

Table of Content

S1. CV and EIS curves of BSA/Ab/O-VMSF/CNNS/GCE to various concentrations of CA15-3

S1. CV and EIS curves of BSA/Ab/O-VMSF/CNNS/GCE to various concentrations of CA15-3

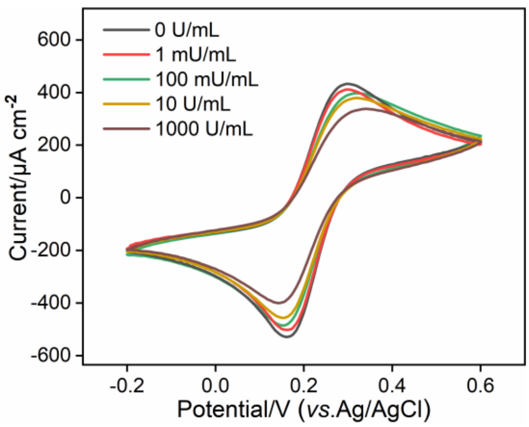

(a)

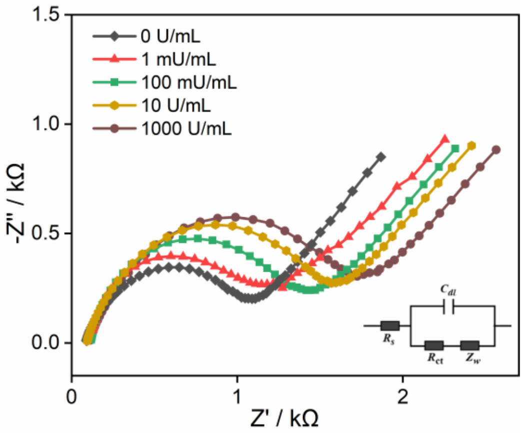

(b)

Figure S1. CV (a) and EIS responses (b) of the prepared BSA/Ab/O-VMSF/CNNS/GCE to various concentrations of CA15-3 in 0.1 M KCl solution containing 2.5 mM $[Fe(CN)_6]^{3-/4-}$. Inset in (b) is the equivalent circuit diagram.
